# Linking bacterial enterotoxins and alpha defensin 5 expansion in the Crohn’s colitis: A new insight into the etiopathogenetic and differentiation triggers driving colonic inflammatory bowel disease

**DOI:** 10.1371/journal.pone.0246393

**Published:** 2021-03-09

**Authors:** Tanu Rana, Olga Y. Korolkova, Girish Rachakonda, Amanda D. Williams, Alexander T. Hawkins, Samuel D. James, Amos M. Sakwe, Nian Hui, Li Wang, Chang Yu, Jeffrey S. Goodwin, Michael G. Izban, Regina S. Offodile, Mary K. Washington, Billy R. Ballard, Duane T. Smoot, Xuan-Zheng Shi, Digna S. Forbes, Anil Shanker, Amosy E. M’Koma

**Affiliations:** 1 Department of Biochemistry, Cancer Biology, Neuroscience and Pharmacology, Meharry Medical College School of Medicine, Nashville, Tennessee, United States of America; 2 Department of Microbiology and Immunology, Meharry Medical College School of Medicine, Nashville, Tennessee, United States of America; 3 Department of Biology, Lipscomb University, Nashville, Tennessee, United States of America; 4 Division of General Surgery, Section of Colon and Rectal Surgery, Vanderbilt University School of Medicine, Nashville, Tennessee, United States of America; 5 Department of Pathology, Anatomy and Cell Biology, Meharry Medical College School of Medicine, Nashville General Hospital, Nashville, Tennessee, United States of America; 6 Department of Pathology, Microbiology, and Immunology, Tennessee Valley Health Systems VA Medical Center, Vanderbilt University School of Medicine, Nashville, Tennessee, United States of America; 7 Department of Biochemistry, Cancer Biology, Neuroscience and Pharmacology, Meharry Medical College School of Graduate Studies and Research, Nashville, Tennessee, United States of America; 8 Department of Biostatistics, Vanderbilt University School of Medicine, Nashville, Tennessee, United States of America; 9 Department of Professional and Medical Education, Meharry Medical College School of Medicine, Nashville General Hospital, Nashville, Tennessee, United States of America; 10 Department of Pathology, Microbiology and Immunology, Vanderbilt University School of Medicine, Nashville, Tennessee, United States of America; 11 Department of Medicine, Meharry Medical College School of Medicine, Nashville General Hospital, Nashville, Tennessee, United States of America; 12 Department of Medicine, University of Texas Medical Branch (UTMB) in Galveston, Galveston, Texas, United States of America; University of Illinois at Chicago, UNITED STATES

## Abstract

Evidence link bacterial enterotoxins to apparent crypt-cell like cells (CCLCs), and Alpha Defensin 5 (*DEFA5*) expansion in the colonic mucosa of Crohn’s colitis disease (CC) patients. These areas of ectopic ileal metaplasia, positive for Paneth cell (PC) markers are consistent with diagnosis of CC. Retrospectively, we: **1.** Identified 21 patients with indeterminate colitis (IC) between 2000–2007 and were reevaluation their final clinical diagnosis in 2014 after a followed-up for mean 8.7±3.7 (range, 4–14) years. Their initial biopsies were analyzed by *DEFA5* bioassay. **2.** Differentiated ulcer-associated cell lineage (UACL) analysis by immunohistochemistry (IHC) of the CC patients, stained for Mucin 6 (MUC6) and *DEFA5*. **3**. Treated human immortalized colonic epithelial cells (NCM460) and colonoids with pure DEFA5 on the secretion of signatures after 24hr. The control colonoids were not treated. **4**. Treated colonoids with/without enterotoxins for 14 days and the spent medium were collected and determined by quantitative expression of *DEFA5*, CCLCs and other biologic signatures. The experiments were repeated twice. Three statistical methods were used: (i) Univariate analysis; (ii) LASSO; and (iii) Elastic net. *DEFA5* bioassay discriminated CC and ulcerative colitis (UC) in a cohort of IC patients with accuracy. A fit logistic model with group CC and UC as the outcome and the DEFA5 as independent variable differentiator with a positive predictive value of 96 percent. IHC staining of CC for MUC6 and *DEFA5* stained in different locations indicating that *DEFA5* is not co-expressed in UACL and is therefore NOT the genesis of CC, rather a secretagogue for specific signature(s) that underlie the distinct crypt pathobiology of CC. Notably, we observed expansion of signatures after *DEFA5* treatment on NCM460 and colonoids cells expressed at different times, intervals, and intensity. These factors are key stem cell niche regulators leading to *DEFA5* secreting CCLCs differentiation ‘the colonic ectopy ileal metaplasia formation’ conspicuously of pathogenic importance in CC.

## Introduction

Predominantly colonic inflammatory bowel disease (IBD), which encompasses ulcerative colitis (UC) and colonic Crohn’s colitis (CC), are two highly heterogeneous chronic relapsing and remitting gastrointestinal disorders in the colon [1–4]. Therefore, understanding the biomolecules and different cellular mechanisms driving IBD heterogeneity is key to the drug inhibitor development to improving patient care. We recently reported that the antimicrobial peptide alpha defensin 5 (*DEFA5*, also abbreviated as HD5) of human Paneth cell-like cells, apparent crypt-cell-like cells (CCLCs), is a useful biomarker accurately differentiating UC from CC. We applied it in a cohort of patients with indeterminate colitis (IC) resulting in highly accurate determinations for authentic UC or CC many times greater than reported experiences at any academic IBD centers [5]. The diagnosis may be made at a patient’s first IBD clinic visit using endoscopy biopsy mitigating diagnostic delay in colon IBD.

Although IBD patients are inflammation-prone, the notion that UC and CC are histologically different and that *DEFA5* and/or specific proinflammatory signatures (cytokines, chemokines, and growth factors) play a major role in the etiopathogenesis trigger of these disease subtypes is still not well established. Our experiments provide evidence suggesting that high levels of *DEFA5* in CC colectomy samples arise from aberrant ectopic ileal metaplastic CCLCs [5]. Whether there is a correlation with our findings observed in sera from UC and CC patients contain high levels of IBD subtype-specific signatures [6] remains to be determined and the mechanisms producing these signatures in the distinct IBD subtypes remain poorly understood. We evaluated *DEFA5* treatment as a secretagogue, on immortalized colonic epithelial cells and colonoids to determine which subtypes of signatures are secreted that may be responsible for IBD inflammation and differentiation.

First, we wanted to identify the origin of *DEFA5* within the colon. Second, we wanted to determine if we can develop *DEFA5*, as a fit logistic model as the independent variable using proteomics and bioinformatics technologies on IC, CC, and UC colectomy tissue samples. Third, we used human colonoids to allow for more facile and robust exploration of cellular physiologic modeling of intestinal response to stimuli as compared to immortalized colonic epithelial cells. Due to the lack of optimal *in vitro* culture systems and the absence of *de facto* animal models for CC, we treated immortalized colonic epithelial cells and colonoids with purified *DEFA5* and bacterial enterotoxins to test biological signature formation.

## Authentication of key biological and/or chemical sources

An important biological resource are colonoids and Immortalized colonic epithelial cell lines. These biologicals were utilized for the cell and organ model experiments. Human colonoids in vitro organ-like culture system is rapidly becoming the new gold standard for investigation of intestinal stem cell biology and epithelial cell physiology.

### Colonoids

Colonoid cultures using the well-established and successful protocols in M’Koma Lab was used. Colonoids were purchased from Cellesce Limited (Medicenter, Cardiff, United Kingdom [UK]). The original colonoids were licensed from the Director of Organoid Biology, Professor Trevor Dale’s Laboratory at Cardiff University, UK. Cellesce (Cardiff, UK) (https://pubmed.ncbi.nlm.nih.gov/31033964) is a Biotrech company that has a patented bioprocessing technology for the growing and expansion of commercialized organoid models enabling drug discovery and genetics research [7].

Human colonoids functionally recapitulate normal intestinal physiology and pathophysiology and are three-dimensional (3D) in-vitro-grown cell culture with near-native microanatomy that arise from self-organizing mammalian pluripotent or adult stem cells. Recent advances in the 3D culture of isolated intestinal crypts have enabled the generation of human gastrointestinal epithelial organoids. Gastrointestinal organoids recapitulate the human in vivo physiology because of all the intestinal epithelial cell types that differentiated and proliferated from tissue resident stem cells. Thus far, gastrointestinal organoids have been extensively used for generating gastrointestinal disease.

### Immortalized colonic epithelial cells

The immortalized colonic epithelial cells, (NCM460) is a human adult, normal-colon epithelium-derived cell lines, derived from the normal colon of a 68-year-old Hispanic male [8]. The NCM460 was received by a Material Transfer Agreement with INCELL Corporation (San Antonio, Texas, USA). The cells express cytokeratins, villin, and other colonic epithelial cell antigens associated with other cell types. Some of the cells are positive for mucin synthesis. They do not grow in soft agar and are non-tumorigenic. They have fastidious growth requirements and must be maintained in INCELL’s enriched M3:10™ medium for long-term in vitro culture with maintenance of an appropriate phenotype.). Human colon epithelial cells, NCM460 (https://www.incell.com/product/ncm460d-cell-line) were derived from the normal colon mucosa from a 68-year-old Hispanic male individual selected for *in vitro* growth [8]. This cell-line was not infected or transfected with any exogenous genetic information. We were interested to use this cell-line to find out whether the release of UC or CC associated cytokines is triggered by the same mechanisms that stimulate DEFA5 secretion by PCs. It is also not clear whether the effects of *DEFA5* and CC-associated cytokines are synergistic in protecting the colon epithelium. To study the mechanisms underlying the secretion of *DEFA5*, as an alternative to the colonoids, we maintained the normal epithelial immortalized colonic epithelial cells, NCM460 in 2D or 3D cultures and assessed the effects of CC and UC-associated signatures/cytokines on the secretion of DEFA5, and downstream signaling effectors. The cells express cytokeratins, villin, and other colonic epithelial cell antigens associated with other cell types. Some of the cells are positive for mucin synthesis. They do not grow in soft agar and are non-tumorigenic. They have fastidious growth requirements and must be maintained in INCELL’s enriched M3:10™ medium for long-term in vitro culture with maintenance of an appropriate phenotype.

#### Reagents, antibodies, and kits

The supplies (reagents, antibodies, and kits) used in experiments are herewith summarized in **Table 1**.

**Table 1:** List of items used in experiments. Reagents, antibodies, kits, vendors and catalog numbers.

	Item Description	Vendor	Catalog No.
1	M3:Base TM	Incell Corporation	M300A-500
**2**	FBS	Invitrogen	16000036
**3**	PBS	Invitrogen	10010023
**4**	Human Cytokine Membrane Antibody Array	Abcam	Ab133998
**5**	Recombinant Human Defensin, Alpha (DEFA5) 5 20ug	Origene	TP310219
**6**	IL-6	Biolegend	430508
**7**	TFN-α	Biolegend	430201
**8**	DMEM-F12	Invitrogen	11320033
**9**	Lipopolysaccharide (LPS)	Millipore Sigma	L7770
**10**	Lipoteichoic acid (LTA)	Millipore Sigma	14015
**11**	Staphylococcal aureus enterotoxin (SAE)	Millipore Sigma	CAS No. 11100-45-1

### Immunohistochemistry and western blotting

Five colon tissue protein extracts and staining of DEFA5 per disease by IHC was done as previously described [9,10]. Quantification of HD5 staining was analyzed manually by microscopy and automatically quantified using Nikon’s Eclipse Ti microscope with built-in NEARAS [10].

Western blot was used to assess any differences in *DEFA5* protein levels. Protein was extracted from a minimum of 10 colon biopsy samples each from UC, CC, and IC. Whole cell lysates were extracted from full-thickness colon samples using T-PER (Thermo Fisher Scientific) per manufacturer’s protocol. Bradford Assays (Bio-Rad) were run to determine protein concentration, and protein was loaded onto a 4–20% SDS-PAGE tris/glycine gel (Bio-Rad). Proteins were transferred to PVDF (Bio-Rad), and Western blots for DEFA5 and β-actin loading control were performed with primary and secondary antibodies (Santa Cruz, Dallas, TX) per manufacturer’s protocol. Blots were visualized with Opti-4CN colorimetric detection kit (Bio-Rad) and imaged with ChemiDoc XRS+ imaging system (Bio-Rad). Band intensities were measured, and data analysis performed with Image Lab Software (Bio-Rad).

ELISA, cytokines assays, lysozyme ELISA kit and enterotoxins (LPS, LTA, SAE) was purchased from R&D Systems, BD Biosciences (www.bdbiosciences.com), Cell Technology LTD (www.immunospot.com), Abcam (http://www.abcam.com), ThermoFisher Scientific and Sigma-Aldrich (www.sigmaaldrich.com). Specificity of antibodies was tested using positive and negative controls provided by companies and available in the laboratories of PIs in the respective assays.

## Materials and methods

Proteomics, microarray, immunohistochemistry, Western blot, Nikon Element Advanced Research Analysis Software (NEARAS), bioinformatics, and NanoString nCounter® technologies were used and compared with qRT-PCR as previously described [5,11,12].

## Ethical consideration and clinical samples

### Colectomy tissue samples from IBD patients

In order to carryout proteomic and gene tissue profiling of differentially expressed proteins/genes in IBD, we first sought ethical approval from the Meharry Medical College (IRB file #: 100916AM206) and Vanderbilt University Medical Center (IRB file #s: 080898 and 100581) Institutional Review Boards. Informed consent was provided, and patient participation in the study was voluntary. Samples were obtained from the NIH-funded Digestive Disease Research Center (PI: David A. Schwartz), the Vanderbilt Gastrointestinal Biospecimen Repository, and the Cooperative Human Tissue Network at Vanderbilt University Medical Center (PI: Mary K. Washington), in Collaboration with the Meharry Medical College Human Tissue Acquisition Shared Resource Core (PI: Billy R. Ballard) [11,12]. How discovery of *DEFA5* as a potential diagnostic biomarker for determining whether a patient suffering from IBD has UC or CC is previously described [5]. Twenty-one (#21) de-identified adult patient samples were identified. These samples comprised of endoscopy pathology biopsy tissues with inconclusive IC diagnosis at Vanderbilt University Medical Center (VUMC) between 2000 and 2007, and were reevaluated for disease course in 2014 (after a mean surveillance follow-up, 8.7±3.7 (range, 4–14) years, to identify the rates of diagnosis resolution to clinically authentic CC or UC. Diagnosis of each patient was determined based on standard clinical and pathologic features as previously described [13,14] by three gastrointestinal pathologists blinded to clinical diagnosis reconciled and confirmed colitis diagnosis for each patient and represented a consensus among treating physicians. Patients who clinically did or did not changed and maintained the IC diagnosis were tested simultaneously via IHC and NEARAS for *DEFA5* quantitative cell staining to determine if *DEFA5* could be used to identify CC *versus* UC and IC cohort into UC or CC. For each selected sample, medical records data on patient demographics, medical and surveillance of endoscopy and clinical findings history were retrieved and reviewed retrospectively. Samples included in all experiments were taken from various parts of the colon; all inflamed tissue unless otherwise indicated. The availability of a detailed IBD patient database registry at VUMC made chart review and follow-up surveillance possible.

## Cytokines assay

### Immortalized colonic epithelial cells culture and treatment with *DEFA5*

To establish the dose for our experiments we treated Immortalized colonic epithelial cells (NCM 460) with *DEFA5* at 3 different concentrations (5, 10 and 15 μM), collected the supernatant at 6 hr. and 24 hr. and ran ELISA in triplicates for IL 6 and TNF-α (Bioligand). After looking at the results, 10 μM *DEFA5* was used in all future experiments.

NCM 460 were received by a Material Transfer Agreement from INCELL Corporation (San Antonio, Texas, USA) [8]. NCM 460 cells (1x10^6^ cells) were plated in a 100 mm culture dish prior to starting the experiment. At the end of the treatment period, the cell culture supernatant was collected and frozen at –80°C until supernatants from all the treatment time-points were collected. Abcam Cytokine Antibody Array (catalog no. ab133998) was used for the simultaneous detection of multiple cytokines following the manufacturer’s protocol. Briefly, the cytokine array membranes were blocked by incubating with 2 ml 1X blocking buffer at room temperature (RT) for 30 min. Blocking buffer was aspirated, and undiluted cell culture supernatants (1 ml) were added to each well and incubated overnight at 4°C. After sample incubation, membranes were thoroughly washed and incubated with 1 ml of 1X Biotin-Conjugated Anti-Cytokines overnight at 4°C. Next, the membranes were washed again and incubated with 2 ml of 1X HRP-Conjugated Streptavidin for 2 hr. at RT. The membranes were then washed, and the cytokines expressed were detected by chemiluminescence.

### Human colonoids culture and treatment with *DEFA5*

Colonoids have been used previously to study the interaction between intestinal pathogens and intestinal epithelium [15–19]. In this study, human colonoids were purchased from Cellesce Limited (Medicentre, Cardiff, United Kingdom) [7]. Colonoid culture was performed as per instructions provided by the manufacturer (Cellesce, Cardiff, UK). Two thousand (#2000) colonoids per well were plated in a 12-well plate. After 2 days of growth in complete medium, the colonoids were treated with purified Human *DEFA5* (5 ug/ml) for 6 hr. and 24 hr. The control colonoids were not treated. At the end of the treatment period, the colonoids culture supernatant was collected and frozen at –80°C until supernatants from all the treatment time-points were collected. Abcam Cytokine Antibody Array (catalog no. ab133998) was used for the simultaneous detection of multiple cytokines following the manufacturer’s protocol. Briefly, the cytokine array membranes were blocked by incubating with 2 mL 1X blocking buffer at room temperature (RT) for 30 min. Blocking buffer was aspirated and undiluted cell culture supernatants (1ml) were added to each well and incubated overnight at 4°C. After sample incubation, membranes were thoroughly washed and incubated with 1ml of 1X Biotin-Conjugated Anti-Cytokines overnight at 4°C. Then the membranes were washed again and incubated with 2 ml of 1X HRP-Conjugated Streptavidin for 2 hours at room temperature. The membranes were then washed, and the cytokines expressed were detected by chemiluminescence.

### Human colonoids culture and treatment with bacterial enterotoxins

Colonoid culture was performed as per instructions provided by the manufacturer (Cellesce, UK). Two thousand colonoids per well were plated in a 12-well plate. After 2 days of growth in complete medium, the colonoids was treated with enterotoxin following a time course for up to 2 wks. At each time point, we collected both the colonoids and the culture supernatants. Then, we determined the expression and/or secretion of *DEFA5* by ELISA. Untreated colonoids served as control. We also seeded the colonoids at low density in triplicates and allow them to attach overnight. We then treated one set with enterotoxin and leave the other set untreated. We allow the colonoids to form colonies and then, we stained the colonies with anti-DEFA5 antibody to determine if the enterotoxin treatment leads to differentiation into CCLCs. We then determined the expression of *DEFA5* and the secretion of DEFA5-induced signatures (cytokines/chemokines/growth factors) by ELISA. At the end of the treatment period, the colonoids culture supernatant was collected and frozen at –80°C until supernatants from all the treatment time-points were collected. Equal numbers of colonoids were treated with/without enterotoxins (LPS, catalog no. L7770, concentrations—1μg/ml); LTA, catalog no L4015, concentrations– 20 μg/ml; and SAE, CAS Number 11100-45-1, concentrations—1 μg/ml) following a time course for up to 14 days. The experiments were repeated twice. At each time point, we collected both the colonoids and the spent medium and determined the expression and/or secretion of *DEFA5* by ELISA and IHC staining for lysosomes. We also seeded the colonoids at low density in triplicate, allowed to attach overnight, and then treated one set with enterotoxins and the other set without and allowed the colonoids to form colonies for up to 14 days. Colonies were stained with anti-*DEFA5* antibodies to determine whether: (1) the treatment lead to differentiation into CCLCs; and (2) the expression and/or secretion of *DEFA5* by ELISA, as well as other signatures, occurred. For colonoid experiments with DEF5A duplicates were used, we only had enough colonoids to do duplicates. The catalog numbers: IL 6—Biolegend Catalog no 430508, TNF-a—Biolegend Catalog no 430201, and DEFA5—Origene Catalog no TP310219. We have indicated these changes in the methods section of the manuscript.

### Lysozyme assay procedure

The assay was performed using the lysozyme ELISA kit from Abcam (catalog no. ab108880) per manufacturer’s instructions. Briefly, 50 μl of the standards and samples were pipetted in appropriate wells of the 96-well plate and incubated at RT for 2 hr. Next, the plate was washed five times with 200 μl of the 1X wash buffer, and 50 μl of the biotinylated lysozyme antibody was added to each well and incubated for 1 hr. at RT. The plate was again washed five times with wash buffer, and 50 μl of 1X SP conjugate was added to each well. The plate was again incubated for 30 min at RT. After washing the SP conjugate, 50 μl of chromogen substrate was added to each well, and the plate was incubated for another 20 min. The reaction was stopped by adding 50 μl of stop solution to each well, and the plate was read at 450 nm. The standard curve was generated from the obtained results, and the concentration of lysozyme in unknown samples was calculated using the standard curve.

### *DEFA5* association with ulcer-associated cell lineage (UACL)?

We examined the association between injury to the mucosae of the IBD colon and emergence of UACL and/or CCLCs as an active reparative and/or defensive response to ulceration and/or enterotoxins. We used mild disease activity of CC formalin-fixed paraffin-embedded (FFPE) tissue sections for MUC6 *vs*. *DEFA5* IHC staining. Tissue IHC for *DEFA5* detection antibody was accomplished as previously described [5] and for Mucin 6 (CLH5: sC-33668) IHC was done according to the instructions provided by the manufacturer (Santa Cruz Biotech, Inc).

### Bacterial toxins information

#### Lipopolysaccharide (LPS)

LPS was purchased from Millipore sigma (product # L7770). The product is soluble in water (5 mg/ml) or cell culture medium (1 mg/ml) yielding a hazy, faint yellow solution. A more concentrated, though still hazy, solution (20 mg/ml) has been achieved in aqueous saline after vortexing and warming to 70°C-80°C. For cell culture use, LPS was reconstituted by adding 1 μg/ml of sterile balanced salt solution or cell culture medium to a vial (1 mg) and swirling gently until the powder dissolved. Solutions were further diluted to the desired working concentration with additional sterile balanced-salt solutions or cell culture media. LPS was expressed in immortalized colonic epithelial cells and colonoids as a reference material in the development of LPS detection systems.

#### Lipoteichoic acid (LTA)

Lipoteichoic acid was purchased from Millipore sigma (product number L4015, CAS number 56411-57-5). Immortalized colonic epithelial cells and colonoids were treatment with LTA at the concentration of 20 μg/ml for 14 days. *DEFA5* expression was monitored and analyzed using ELISA and histological studies, respectively. *DEFA5* expressed in immortalized colonic epithelial cells and colonoids as a reference material in the development of CCLCs detection systems.

#### Staphylococcal aureus enterotoxin (SAE)

Pure SAE was purchased from Millipore sigma (CAS number 11100-45-1 MDL no. MFCD00082017) was used as a superantigen and immune system activator. Immortalized colonic epithelial cells and colonoids were treatment with SAE at the concentration of 1 μm/ml for the period of 14 days. *DEFA5* expression was monitored and analyzed using ELISA and histological studies, respectively.

#### Statistics

To analyzed *DEFA5* bioassay could be used to mitigate IC into UC and CC, three statistical methods were used to find most distinguishing features between the two-diseases including (i) Univariate analysis: Mann Whitney U test between two groups for each peak and adjust *p*-values by False Discovery Rate (FDR); (ii) LASSO; and, (iii) Elastic net. Since the scale of all measures were small, we multiplied all values by 10,000 as a scale-up of the small value from the original reading for the ease of mathematical treatment. It is equivalent to the effect of a unit change for the same measurement, like turning a measurement of 0.0005 Kg to 500 mg through multiplying it by 1,000,000.

To avoid potential bias in the study and to evaluate the generalizability of the results we followed “The STARD (Standards for Reporting of Diagnostic Accuracy Studies)” recommendation [20] to improve the completeness and transparency of reports of diagnostic accuracy studies. All updated STARD materials, including the checklist, were followed at http://www.equator-network.org/reporting-guidelines/stard.

The Vanderbilt University Microarray Core Laboratory performed statistical analyses for the microarray [5]. Transcriptome level fold changes and the significance of those changes were calculated using one-way ANOVA with Bonferroni’s correction for multiple comparisons. Significantly changed transcripts were defined as having >2.0-fold expression change from controls and a Benjamini-Hochberg (BH) false discovery rate corrected ANOVA p-value < 0.05 indicated a statistical significance.

Dual staining of *DEFA5* and CCLCs DoubleStain IHC was performed on a Lab Vision autostainer 360 (Thermo fisher) using Abcam’s M&R on human tissue (DAB & AP/Red) staining kit (ab210059, Abcam Biotechnology, Cambridge, UK). The manufacture’s recommended conditions were used with the following modifications. The mouse anti-α-defensin 5 (sc-53997, Santa Cruz Biotechnolgy, Inc, Dallas, TX) and rabbit anti-lysozyme (ab-2408) were used at a 1:50 dilution in OP Quanto antibody Diluent (Thermo Fisher, Waltham, WA). Prior to addition of antibody for 45 minutes, tissues were incubated for 10 min with Utravision hydrogen peroxide block (Thermo Fisher) followed by a 5 min incubation with Ultravisoion Quanto protein block. A single incubation with Permanent Red was used for ileum tissue, whereas two consecutive 10 min permanent Red incubations were performed for colonic tissue. Following hematoxylin counter staining, tissue was exposed to Richard-Allen Scientific Blueing Reagent (Thermo Fisher). Antigen retrieval was performed in 1 mM EDTA pH 8.4, 0.05% Tween 20 for 20 minutes at 98˚C (60˚C preheat/70˚C cool down) using the Labvision PT Module (Thermo Scioentific). Image color deconvolution was performed with Fiji ImageJ 1.51f (http://imagej.nih.gov/ij) using the Fast Red, Fast Blue and DAB built in stain vector plugin.

## Results

**Fig 1:**
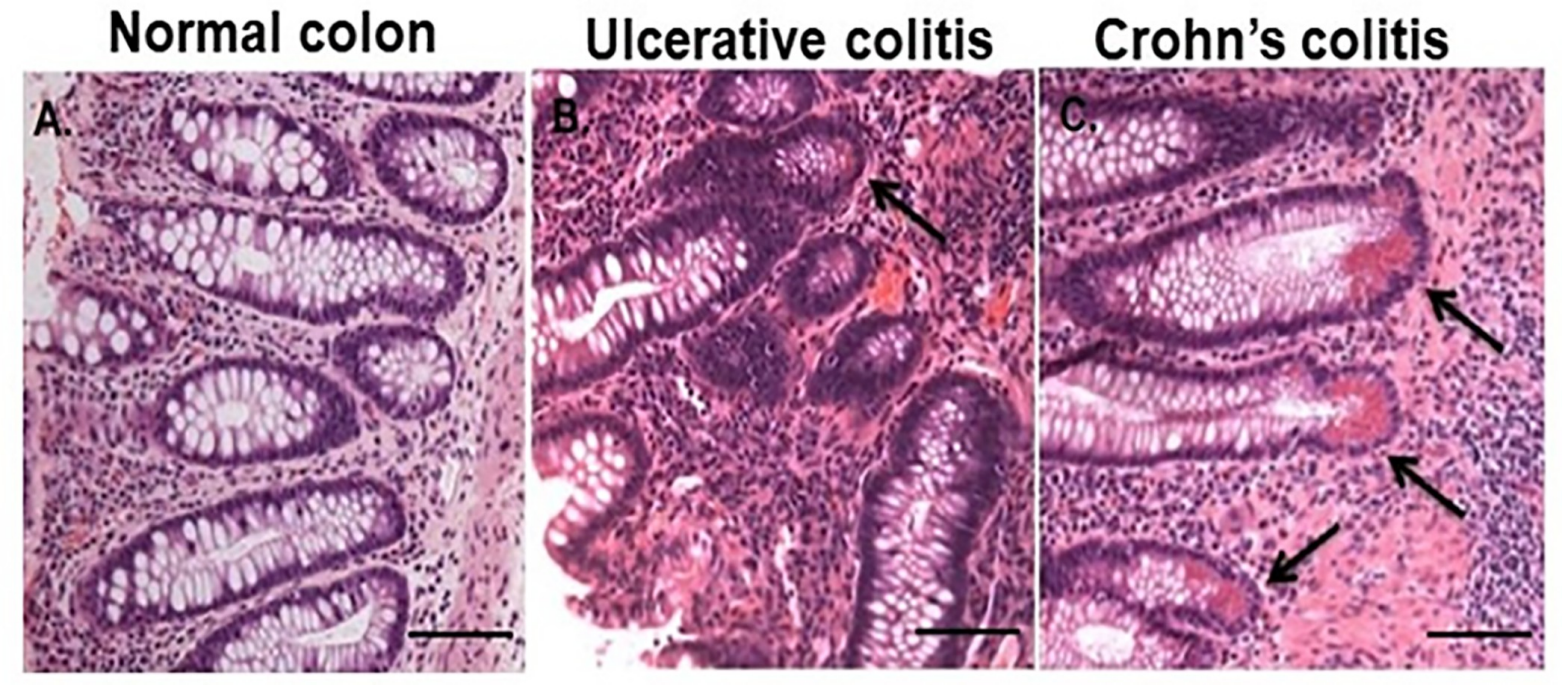
Representative H & E staining of the colon. **A,** Normal**—**normal crypt; **B**, UC–sporadic to no CCLCs even in severe disease activity; and **C**, CC–abundant CCLCs in the crypt base (arrow) seen even in mild disease activity.

Representative Hematoxylin and Eosin (H & E) staining of the colon from PPFE tissue sections from normal, UC and CC depict disparate quantitative levels of CCLCs in the colonic crypt, **Fig 1A–1C**. The CCLCs originate directly from adjacent crypt stem cells. The level of CCLCs expansion did not depend on the location in the colon but severity of the disease. Prevalence of CCLCs was segmental and higher in areas with stricture formation. The role played by precursors of secretory lineages, and by CCLCs, in the generative response to colonic crypt damage is of interest and key to the drug inhibitor development.

**Fig 2:**
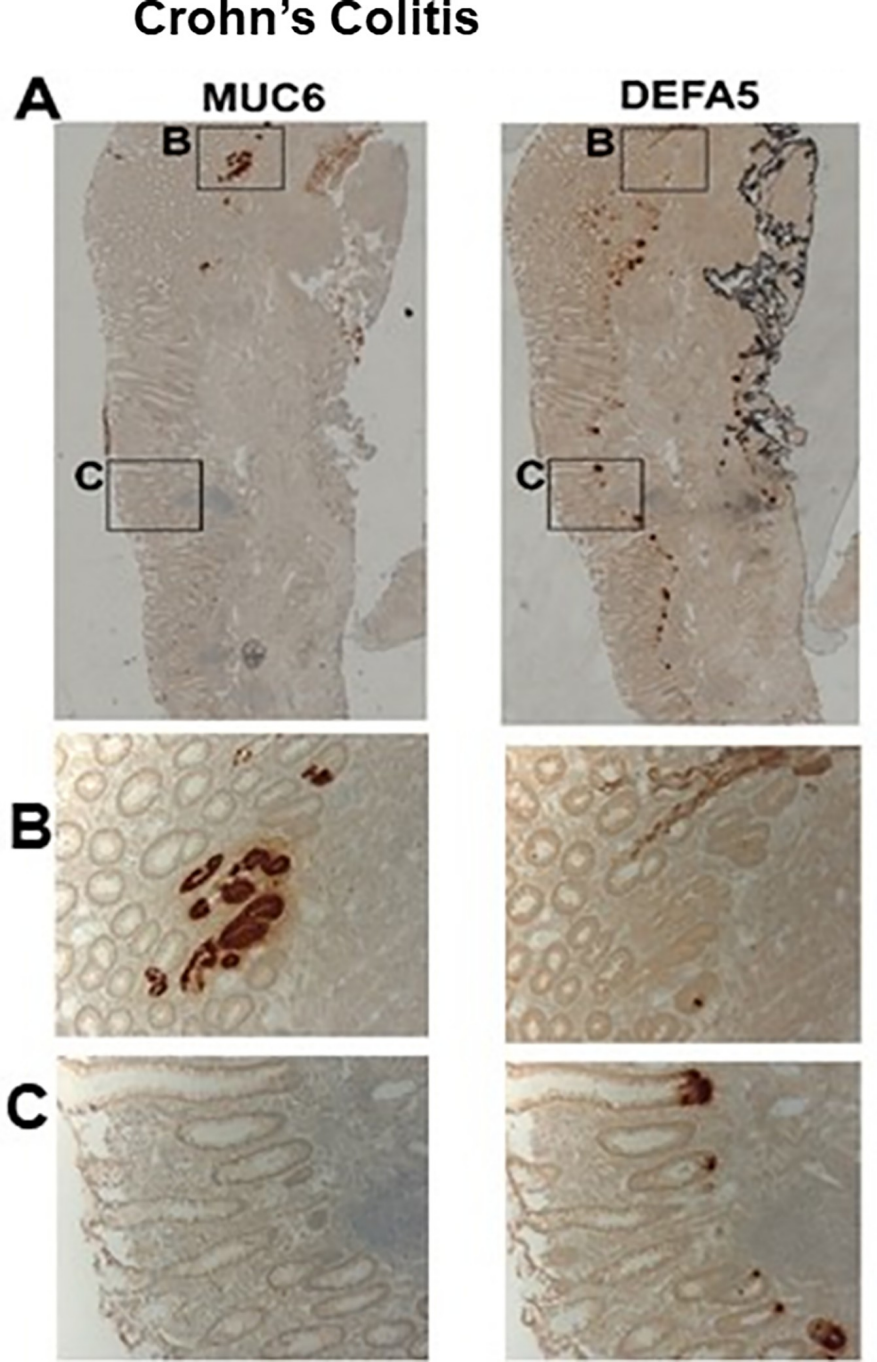
Immunohistochemical detection of CCLCs marker (positive for Paneth cell) *DEFA5* and UACL marker MUC6 in the intestinal mucosa of colonic CC. Seral adjacent tissue sections were stained with MUC6 (left column) and *DEFA5* (right column) on diseased colon from Crohn’s patients. Full tissue section (row A) and enlarged regions associated with MUC6 (box B) or *DEFA5* (box C) demonstrate that DEFA5 expression within the apparent CCLCs is not localized within the UACL region of the diseased colon.

Representative IHC staining of CC colectomy tissue for MUC6 and *DEFA5* revealed differing locations, meaning that CCLCs-secreted *DEFA5* is not co-expressed in UACL (showed crypt-restricted proliferation and commitment to CCLC lineage). This observation demonstrates that *DEFA5* and lysozyme are not expressed in UACL but are in the colonic epithelium lining crypt areas of CC patients and their stromal cell-adjacent normal indicating that the high *DEFA5* levels in CC patient samples arise from CCLCs (**Fig 2A and 2B**). In this light, the presence of *DEFA5* in areas of the colonic mucosa with aberrant expression of CCLCs identifies an ectopic ileal metaplasia (positive for Paneth cell markers) that is consistent with the diagnosis of CC. Apparent CCLCs share their differentiation program with stem cells, but the pattern of eventual cell renewal is in the ectopic ileal crypt of the colon.

### Circumventing IC into authentic CC or UC

**Fig 3:**
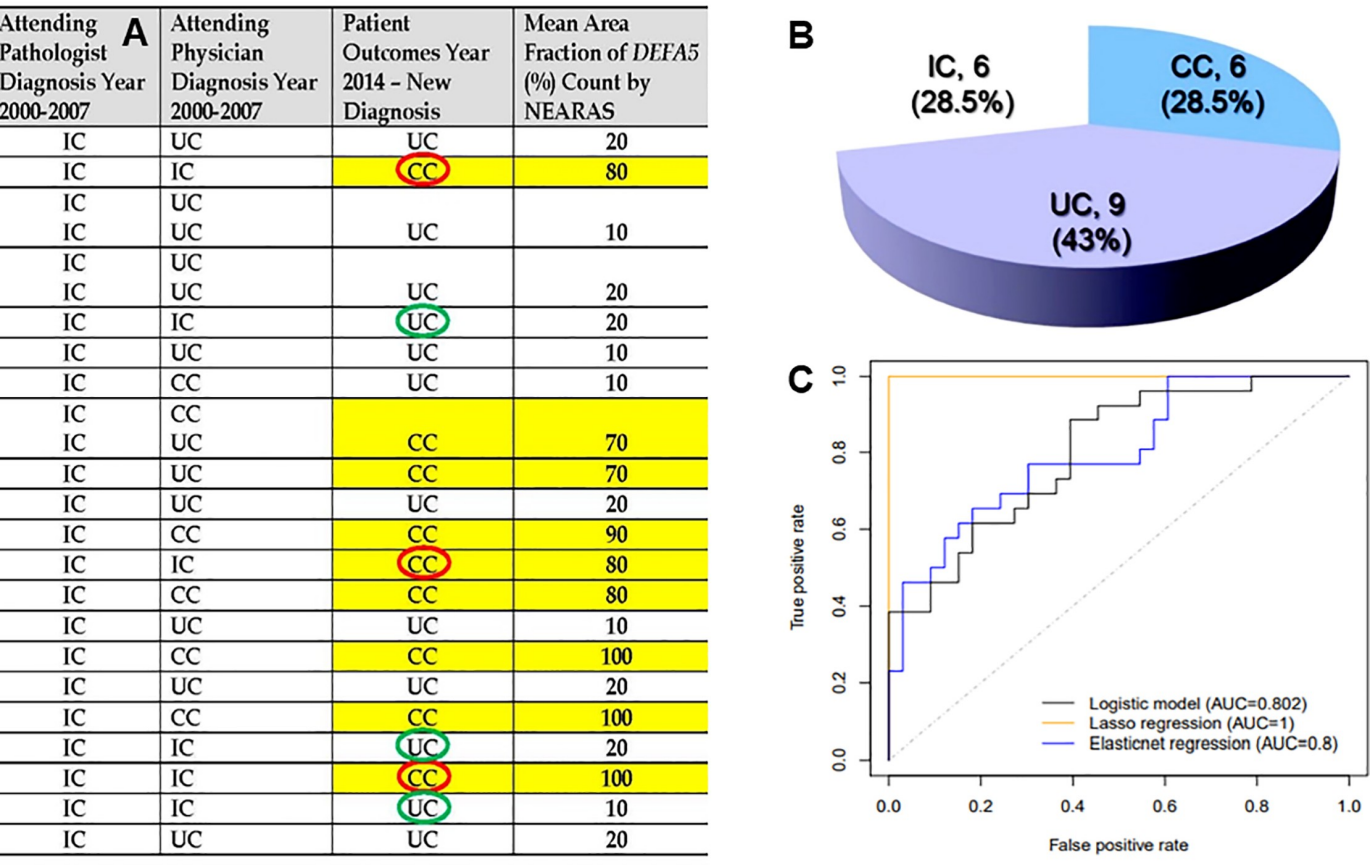
Multivariable logistic regression model for probability of being CC. **A**, twenty-one (#21) IC patients were followed for 8.7±3.7 (range, 4–14) years. Although most IC resolved to UC or CC, but the mean diagnostic delay was 7 (range, 4–14) years. 28.5% of the patients could still not be diagnosed into authentic UC or CC. The mean area fraction of *DEFA5* count (%) by NEARAS IHC staining agrees with final diagnostic outcome of every IC patient samples tested. **B**, Clinical data of these 21 IC patients who correlate with 3A data could have been diagnosed using *DEFA5* immunoreactivity during the first endoscopy biopsy to avoid diagnosis delay. **C**, Bioinformatic data depicting a fit logistic model with group CC *vs*. UC (in 3A) as the outcome and *DEFA5* as independent variable discriminator. The R2 of the model fit is 1 and area under the ROC curve is 1. The area under the ROC curve was not plotted since it will look similar as the orange curve in ROC plot. Three statistical methods were used: (1) Univariate analysis: Mann Whitney U test between two groups for each peak, adjusting *p*-values by FDR; (2) LASSO; and (3) Elastic net. As the scale of all measures were small, we multiplied all values by 10,000.

To determine if *DEFA5* could be used to assess whether IC patients could be delineated into a diagnosis of either UC or CC instantly, we assessed levels of *DEFA5* in surgical pathology colon samples using IHC. in all these 21 IC patients, in each instance of a final diagnosis of CC, *DEFA5* high NEARAS counts agreed with that diagnosis, **Fig 3A & 3B**. We also found that when the 6 patients that remained clinically and histologically inconclusive (by an attending gastroenterologist and by 3 blinded pathologists) and retained unchanged IC diagnosis were analyzed using *DEFA5* IHC NEARAS profile counts, **Fig 3A**. Three patients (n = 3) showed high *DEFA5* count and agreed with the final diagnosis of CC (red front circle) and another 3 patients had low *DEFA5* count and were in agreement with the final diagnosis of UC (green front circle). Further, bioinformatically, a fit logistic model depicts group CC *vs*. UC as the outcome and *DEFA5* as independent variable discriminator biomarker, **Fig 3C**. The R2 of the model fit is 1 and area under the ROC curve is 1. The area under the ROC curve was not plotted since it will look similar as the orange curve in ROC plot.

### Bacterial enterotoxins treatment on immortalized colonic epithelial cells (NMC460) and colonoids

**Fig 4:**
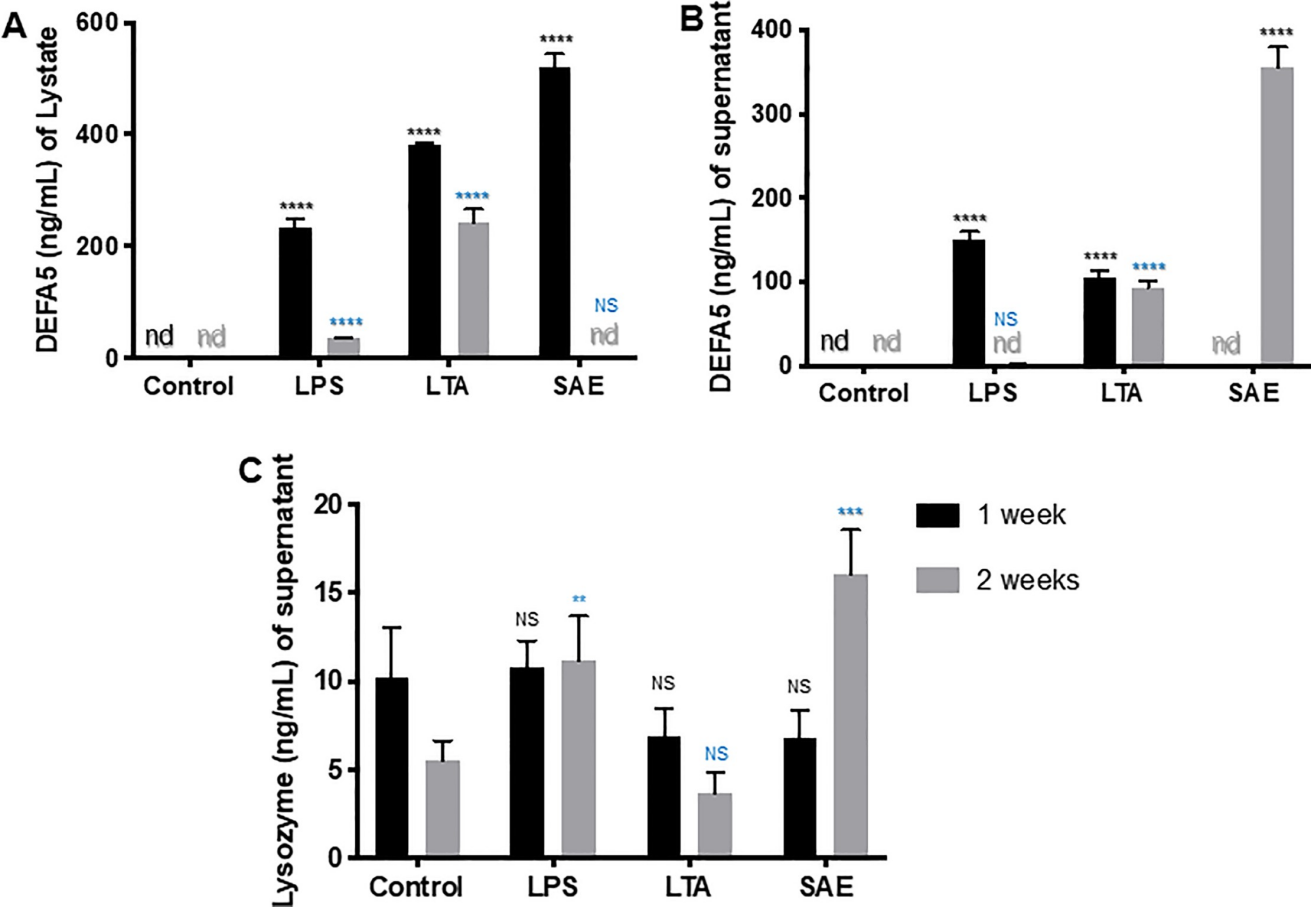
Treatment of colonoids with bacterial enterotoxins. **A–B,** Equal numbers of colonoids were treated with/without enterotoxins (LPS, LTA, SAE) following a time course of up to 14 days. At each time point, we collected both the colonoids and the spent medium and determined the expression and/or secretion of *DEFA5* by ELISA and IHC staining for lysozymes. We also seeded the colonoids at low density in triplicate, allowed to attach overnight, and then treated one set with enterotoxins and the other set without. We allowed the colonoids to form colonies for 14 days. Colonies were stained with anti-*DEFA5* to determine whether the treatment led to differentiation into apparent CCLCs. The expression and/or secretion of *DEFA5* and proinflammatory cytokines, etc. was determined by ELISA. **C,** Lysozyme assay: The assay was performed using lysozyme ELISA kit firm abcam (catalog no. ab108880 as per manufacturer’s instructions.

We observed that *DEFA5* was significantly higher in lysate during the end of the first week of treatment with LPS and LTA compared with controls (**Fig 4A)** and correlated to enterotoxins and duration of treatment, *P< 0*.*0001*. The reverse was true in the supernatant (**Fig 4B**). During the second week of SAE treatment, *DEFA5* in supernatant was increased 3-fold, *P< 0*.*0001* and was not detected (nd) in the lysate (**Fig 4B**). This suggests that immune stimuli are a stem cell niche precursor for *DEFA5*-secreting CCLCs expansion. Lysozyme analysis of the supernatant tested positive, an indication that CCLCs have differentiated (**Fig 4C**). In patients with IBD, this hypothesizes that bacteria are enriched at the inflamed colonic mucosal surface. The complex relationship between microbial distribution and its influence on the host has yet to be elucidated. In that light, we recently developed colonoid culture to reveal the mechanistic basis for genetic susceptibility to CC, which will also provide a diagnostic platform for therapeutics, including individualized therapeutics, and understanding the effects of immune and microbial stress on epithelial barrier function.

### *DEFA5* treatment on immortalized colonic epithelial cells and colonoids

**Fig 5:**
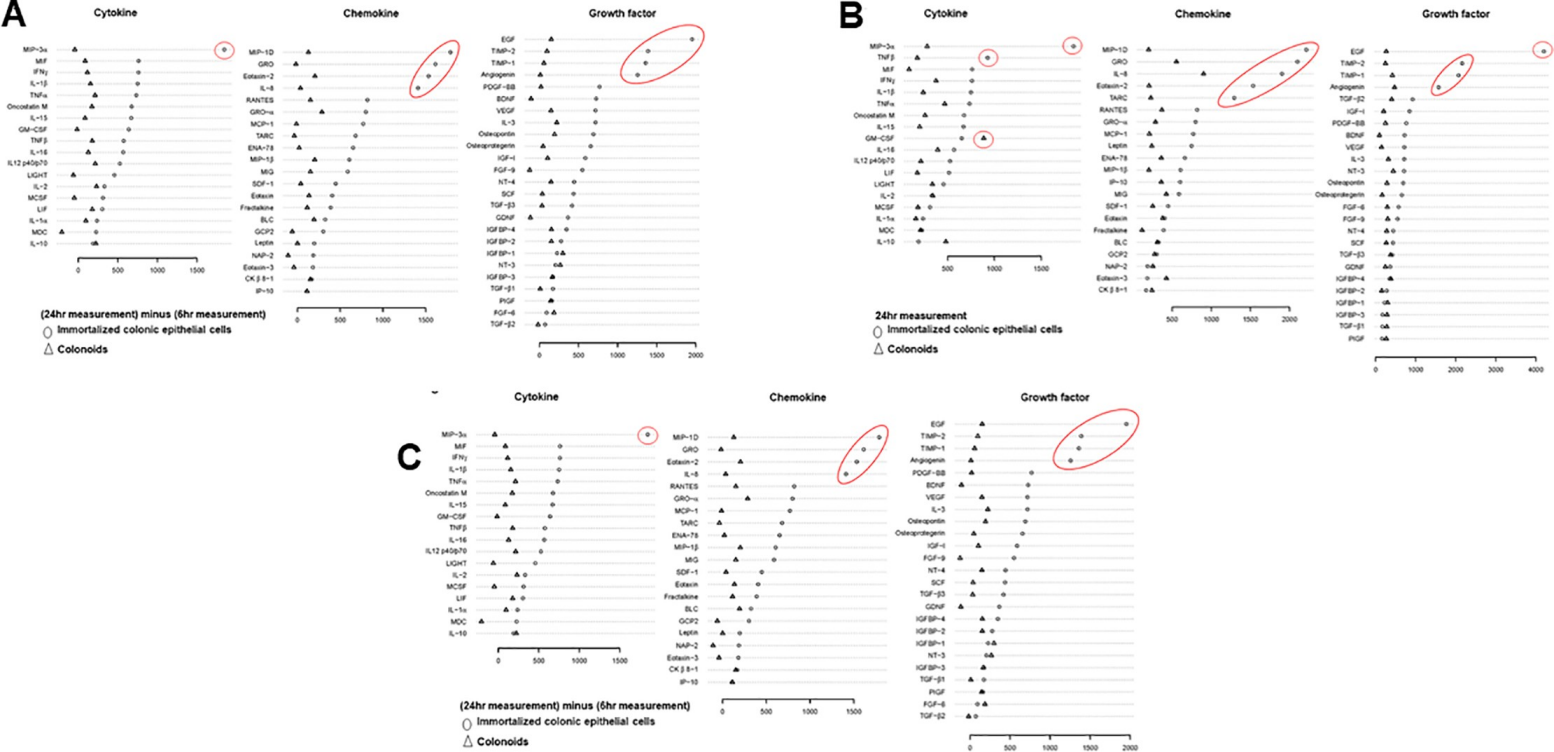
Treatment of immortalized colonic epithelial cells and colonoids with *DEFA5*. We graphically present the data on the signatures for immortalized colonic epithelial cells and colonoids separately. We did not perform statistical tests on these data. Immortalized colonic epithelial cells (NCM 460) were received by a Material Transfer Agreement from INCELL Corporation (San Antonio, Texas, USA) [8]. NCM 460 cells (1x10^6^ cells) were plated in a 100 mm culture dish prior to starting the experiment. At the end of the treatment period, the cell culture supernatant was collected and frozen at –80°C until supernatants from all the treatment time-points were collected. Abcam Cytokine Antibody Array (catalog no. ab133998) was used for the simultaneous detection of multiple cytokines following the manufacturer’s protocol. Colonoids were purchased from Cellesce Limited (Medicentre, Cardiff, UK). Organoid culture was performed as per instructions provided by the manufacturer (Cellesce). Two thousand colonoids per well were plated in a 12-well plate. After 2 days of growth in complete medium, the colonoids were treated with purified Human *DEFA5* (5 μg/ml) for 6hr. and 24hr. The control colonoids were not treated. At the end of the treatment period, the colonoids culture supernatant was collected and frozen at –80°C until supernatants from all the treatment time-points were collected. The experiments were repeated twice. At each time point, we collected both the colonoids and the spent medium and determined the expression and/or secretion of *DEFA5* by ELISA and IHC staining for lysozymes. We also seeded the colonoids at low density in triplicate, allowed to attach overnight, and then treated one set with enterotoxins and the other set without and allowed the colonoids to form colonies for up to 14 days. Cytokines, chemokines, and growth factor measurements (signatures) are depicted: **A**, 24hr measurement; **B**, (24hr measurement) minus (6hr measurement) and **C**, (24hr measurement) minus (Control 24hr measurement) respectively.

Immortalized colonic epithelial cell is an epithelial cell monolayer lining of the colon while a colonoid cell is a miniaturized and simplified version of a colon organ produced in vitro in three dimensions that shows realistic micro-anatomy. Treatment of *DEFA5* on immortalized colonic epithelial cells and colonoids induced expression of various subtype signatures (**Fig 5A–5C**). We graphically present the cytokines, chemokines, and growth factors (signatures) measured on immortalized colonic epithelial cells and colonoids at 24 hr. We observed that at 24 hr., certain signatures were disparately secreted between the immortalized colonic epithelial cells and colonoids, specifically MIP-3α, TNF-β, MIF, IFN-γ, and IL-1β; MIP-1D, GRO, IL-8, Eotaxin, TARC; EGF, TIMP-2, TIMP-1, and Angiogenin (**Fig 5A**).

At 24 hr. *vs*. 6 hr. measurement analyses, we further observed a wider range of intensity secretion disparity especially for MIP-3α, MIF, IFN-γ, IL-1β, TNF-α, Oncostatin M, IL-15, GM-CSF, TNF-β, and IL-16; MIP-1D, GRO,Eotaxin-2, IL-8, RANTES, GRO-α, MCP-1, TARC, ENA-78, MIP-1β, MIG, SDF-1; EGF, TIMP-2, TIMP-1, Angiogenin, PDGF-BB, BDNF, VEGF, IL-3, Osteopontin, Osteoprotegerin, IGF-I, and FGF-9 (**Fig 5B**).

Finally, we calculated the cytokines differential expression, chemokines, and growth factors measured on immortalized colonic epithelial cells or colonoids and control at 24 hr. (**Fig 5C**). **Fig 5C** depicts disparity at 24 hr. *vs*. control 24 hr. measurements, MIP-3α, TNF-β, MIF, IL-1β, IL-15; MIP-1D, Exotaxin-2, GRO, TARC, IL-8; EGF, TIMP-2, TIMP-1, and Angiogenin. Among these, GM-CSF (a stem-cell stimulator that works to produce granulocytes and monocytes. It is interesting that this cytokine is increased in an area where we are seeing differentiation of stem cells in IBD. Eotoxin-3, and IL-10 were the most highly secreted in colonoids. This disparate release of inflammatory proteins is intriguing, given the important role of defensins in regulating the immune system.

Our study finds the high expression levels of *DEFA5* in colonoid cells treated with bacterial enterotoxins, which can be found in patients with CC with presence of enriched bacterial enterotoxins. Cytokines differential expression observed in colonoids and their disparate are depicted **Fig 5A–5C**.

## Conclusion

Paneth cell-like cells, the apparent CCLCs stained for MUC6 and *DEFA5* in different locations, meaning that CCLCs-secreted *DEFA5* is not co-expressed in UACL. *DEFA5* is therefore NOT the genesis of CC, rather is an intermediate secretagogue for DEFA5-induced specific signatures that underlie the distinct colonic crypt pathobiology of CC. Ectopic colonic crypt ileal metaplasia formation is therefore of pathogenic importance in CC. In lieu of these robust analytical studies reveal critical insights into the role of *DEFA5* in differentiating the CC subtype of IBD, illuminating *DEFA5* as a potential therapeutic target for CC. Targeting *DEFA5*-mediated pathogenic pathways (immunopathological mechanisms) for drug development would attain histologic and clinical remission that could replace surgery and subsequent recurrences after surgical resection which are 100% in CC, given sufficient time.

## Discussion

With the increasing incidence of medically incurable IBD worldwide, discrimination between CC and UC has become more important but difficult based on biopsies [1,15–19,21]. Clinical information is often helpful, and definitive diagnosis can in most cases, be made upon examination of resection specimens. Nevertheless, preoperative diagnosis is helpful in guiding surgical planning and, in some cases, medical management. The fraction of IC herewith reported is many times greater than reported experiences at academic IBD centers worldwide, means for better diagnosis of IC cases, particularly on biopsy specimens, would be extremely useful. This work depended solely on full thickness colectomy specimens for both discovery (training test) and validation (independent test) studies, but we believe that the use of endoscopy biopsies results would be indifference. Hospital admission rates and costs for IBD show an increasing trend assessed by specific pharmaceutical and disease features [15–19,21–26]. In the US alone, the estimated annual direct treatment costs are greater than $6.8 billion, and indirect costs amount to an additional $5.5 billion [27,28]. There is a need to better understand epithelial disorders of IBD and disease mechanisms that drive its heterogeneity to develop targeted treatment strategies without the adverse effects of systemic immunosuppression [29–34]. From Vanderbilt University, Goldenring, *et al*. examined the association between injury to the mucosae of the GI tract and emergence of metaplastic lineages as an active reparative response to ulceration [35]. The notion that UC and CC are histologically different suggests that *DEFA5*, and/or in association with *DEFA5*-induced signatures, suggests a link between *DEFA5* and bacterial enterotoxins in the pathogenesis and differentiation of IBD [6,36]. It is unknown whether high levels of *DEFA5* alone may be deleterious to intestinal tissues. Our studies depict that colonic epithelium with positive staining *DEFA5* and lysozyme in the injured crypt area of CC patients and their stromal cells adjacent to normal cells indicate that the high *DEFA5* levels in CC colectomy samples arise from ectopic ileal metaplasia Paneth cell-like cells, ACCLCs. A Norwegian scientific team has reported pyloric metaplasia in ileal CD [37], which exhibits a specific elevation pattern of certain genes including MUC6 and lysozyme [37,38]. Our studies on MUC6 and *DEFA5* IHC staining of CC tissues show that *DEFA5* is not co-expressed in UACL (**Fig 1A** and **1B**), consistent with the report from the Norwegian research team [37,38]. These studies could lend mechanistic insight into UC and CC pathogenesis. The role of *DEFA5* and overall CCLCs physiology remains unclear in the development of CC and other gut dysbiosis disorders [39]. We have reported earlier that levels of IFN-γ, IL-6, and IL-7 are higher in sera from CC patients, while levels of CCL11, CXCL1, and TNF-α are higher in sera from UC patients [6]. Further, we reported that *DEFA5* differentiates CC from UC [5]. In addition, *DEFA5* accurately identifies the CC or UC phenotype among IC patient cohorts with a positive predictive value of 96 percent [5]. Finally, we reported that *DEFA5* may be used to determine appropriate patient candidacy for ileal pouch-anal anastomosis (IPAA) surgery and also may predict patients with UC who are likely to transform and convert to *de novo* CD after IPAA surgery [40].

We evaluated the differentiation of CCLCs by treating colonoids with enterotoxins. This investigation of *DEFA5*, comparing the secretion of signatures by immortalized colonic epithelial cells and colonoids, showed large quantities of *DEFA5* in the lysate of colonoid cultures exposed to different toxins during the first week and in the supernatant during the second week of treatment. We also observed evidence of CCLC differentiation in the colonoids using the lysozyme assay test.

We observed various responses from different enterotoxins treatment and *DEFA5* on immortalized colonic epithelial cells vs. colonoids, highlighting several epithelial cells pathways, including an upregulating of innate and adaptive immune pathways; the phenomenon seen in CC and in UC, respectively [41]. These observations indicate possible specific pathways uniquely expressed between UC and CC. These biosignatures could be used as biomarkers for IBD and would be invaluable for developing and improving diagnostics and prognostics in IBD clinics.

In our univariate analysis of IBD, patient sera showed higher levels of IFN-γ, IL-6, and IL-7 in CC than in control samples, as well as higher levels of GRO, eotaxin, and TNF-α in UC than in control samples [6]. We showed that these cytokines indeed have distinct predictive probability for the respective IBD subtype [6]. Interestingly, the higher IL-6 and IFN-γ levels in CC are consistent with a TH1-like course of CD, as both cytokines mediate the acute immune responses and contribute to microphage recruitment and activation.

The pharmacological agents for managing IBD (both UC and CD) include glucocorticoids, immunomodulators [42], and anti-TNF-α biological agents [43,44], as well as inhibitors of molecular pathways [45]. Our team [6] and others [46–51] have observed that TNF-α as well as IFN-γ have a direct effect on barrier function, metabolism, and viability in transformed ileal epithelial cells. How human colonic epithelium responds to these immune mediators as secretagogues is still unknown. Using IBD patient sera, we have shown that the proinflammatory cytokines secreted during IBD may be classified further as inflammatory (IFN-γ and TNF-α) and regulatory (IL-6 and IL-7) cytokines, as well as chemokines (eotaxin, GRO, IL-8) [6]. The proinflammatory cytokines have been shown to increase cellular tight-junction permeability [52–54] and mucosal inflammation in IBD patients [55], thus restoring intestinal permeability in response to anti-TNF therapy [56,57]. Furthermore, these cytokines are important mediators of acute responses and contribute to macrophage recruitment and activation [58,59]. Our data have shown robust evidence supporting presence of *DEFA5* in areas of the colon mucosa with aberrant expression of CCLCs identifies an area of ectopic ileal metaplasia positive for Paneth cell markers that is consistent with the diagnosis of CC. Using multipronged cross-sectional technologies revealed that the expression levels of *DEFA5* was aberrantly 118-fold greater in CC as compared to UC, but its disparity involvement remains obscure. We have demonstrated that, in a patient suffering from IBD can accurately be determined and distinguished molecularly that has UC or CC by examining CCLCs secreted *DEFA5* levels in colon biopsies without delay. Further, *DEFA5* reliably discriminate authentic CC and UC in a cohort of IC patients. Elucidating the underlying mechanisms involved in the regulation of crypt biology of *DEFA5* in CC could open new therapeutic target avenues. This is particularly true that the goals of treatment are mucosal healing, histologic and deep clinical remission. The relevant molecular mechanisms of these signatures to sustain the CC must be analyzed individually using colonic crypt-derived colonoids.

## Supporting information

S1 Fig Outlined pictorial schematic hypothetical flowchart. Of note, a normal colon does not have Paneth cells, **S1A.** *DEFA5* production in the ileal mucosa crypt Paneth cells binds and lyse bacteria. We noticed presence of DEFA5 in the colonic mucosal crypt with ectopic expansion of apparent CCLCs which is positive for Paneth cell markers that is consistent with the diagnosis of CC. A prevailing mode for the genesis of IBD is that the intestinal mucosal barrier is compromised leading to chronic inflammation in individuals with genetic predisposition [60]. We observed aberrant Paneth cell like cell morphology, CCLCs, **S1B** and increased levels of *DEFA5* in the colon of CC patients [12], suggesting a role for CCLCs expansion in the colonic mucosal barrier of CC patients. CCLCs, in CC, as reported by others for about Paneth cells, support the stem cell niche expressing ligands for key pathways that maintain a de-differentiated state [61,62].
(TIF)

S1 Table List of targets from NanoString human inflammatory PCR. Sixteen (#16) inflammatory genes were charged in this subset of samples.
(TIF)

## Abbreviations

IBD: inflammatory bowel disease
IC: Indeterminate colitis
UC: Ulcerative colitis
CC: Crohn’s colitis or CCD = Colonic Crohn’s disease
CD: Crohn’s disease
CI: Crohn’s ileitis
DV: Diverticulitis
CCLCs: Crypt cell-like cells
MUC6: Mucin 6
NEARAS: Nikon Element Advance Research Analysis Software
UACL: ulcer-associated cell lineages
H&E: hematoxylin and eosin
HD5: Human alpha defensin 5
IHC: Immunohistochemistry
Lys: lysozymes
DEFA5: Human alpha defensin 5
ROC: receiver operator characteristic
MMC: Meharry Medical College
VU: Vanderbilt University
VUMC: Vanderbilt University Medical Center
NIHR: National Institute for Health Research
VICTR: Vanderbilt Institute for Clinical and Translational Research
