# Correction: Linking bacterial enterotoxins and alpha defensin 5 expansion in the Crohn’s colitis: A new insight into the etiopathogenetic and differentiation triggers driving colonic inflammatory bowel disease

**DOI:** 10.1371/journal.pone.0304732

**Published:** 2024-05-29

**Authors:** Tanu Rana, Olga Y. Korolkova, Girish Rachakonda, Amanda D. Williams, Alexander T. Hawkins, Samuel D. James, Amos M. Sakwe, Hui Nian, Li Wang, Chang Yu, Jeffrey S. Goodwin, Michael G. Izban, Regina S. Offodile, Mary K. Washington, Billy R. Ballard, Duane T. Smoot, Xuan-Zheng Shi, Digna S. Forbes, Anil Shanker, Amosy E. M’Koma

The eighth author’s name is spelled incorrectly. The correct name is: Hui Nian.

In the Discussion section, there is an error in the twelfth sentence of the first paragraph. The figures referenced should be Fig 2A and 2B. The correct sentence is: “Our studies on MUC6 and *DEFA5* IHC staining of CC tissues show that *DEFA5* is not co-expressed in UACL (Fig 2A and 2B), consistent with the report from the Norwegian research team [37,38].”

There is an error in reference 11. The correct reference is: M’Koma AESE, Seeley EH, Washington MK, Schwartz DA, Muldoon RL, Herline AJ, Wise PE, et al. Proteomic profiling of mucosal and submucosal colonic tissues yields protein signatures that differentiate the inflammatory colitides. Inflamm Bowel Dis 2011;17:875–83. pmid:20806340.
