# Supplementary figures and images for: Linking bacterial enterotoxins and alpha defensin 5 expansion in the Crohn’s colitis: A new insight into the etiopathogenetic and differentiation triggers driving colonic inflammatory bowel disease
Source: PLoS One. 2021 Mar 9;16(3):e0246393. doi: 10.1371/journal.pone.0246393 (PMC7942995; doi:10.1371/journal.pone.0246393)

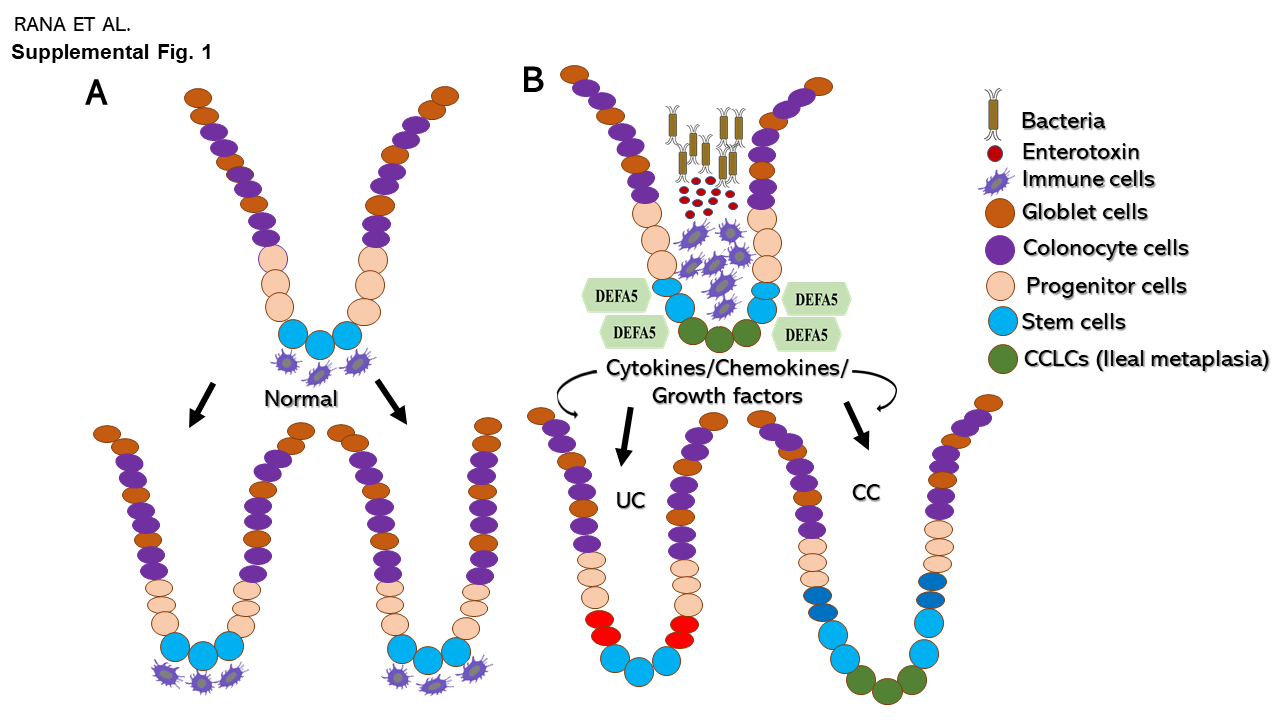

Supplement: S1 Fig — Of note, a normal colon does not have Paneth cells, S1A. DEFA5 production in the ileal mucosa crypt Paneth cells binds and lyse bacteria. We noticed presence of DEFA5 in the colonic mucosal crypt with ectopic expansion of apparent CCLCs which is positive for Paneth cell markers that is consistent with the diagnosis of CC. A prevailing mode for the genesis of IBD is that the intestinal mucosal barrier is compromised leading to chronic inflammation in individuals with genetic predisposition [60]. We observed aberrant Paneth cell like cell morphology, CCLCs, S1B and increased levels of DEFA5 in the colon of CC patients [12], suggesting a role for CCLCs expansion in the colonic mucosal barrier of CC patients. CCLCs, in CC, as reported by others for about Paneth cells, support the stem cell niche expressing ligands for key pathways that maintain a de-differentiated state [61,62]. (TIF) [file pone.0246393.s001.tif]

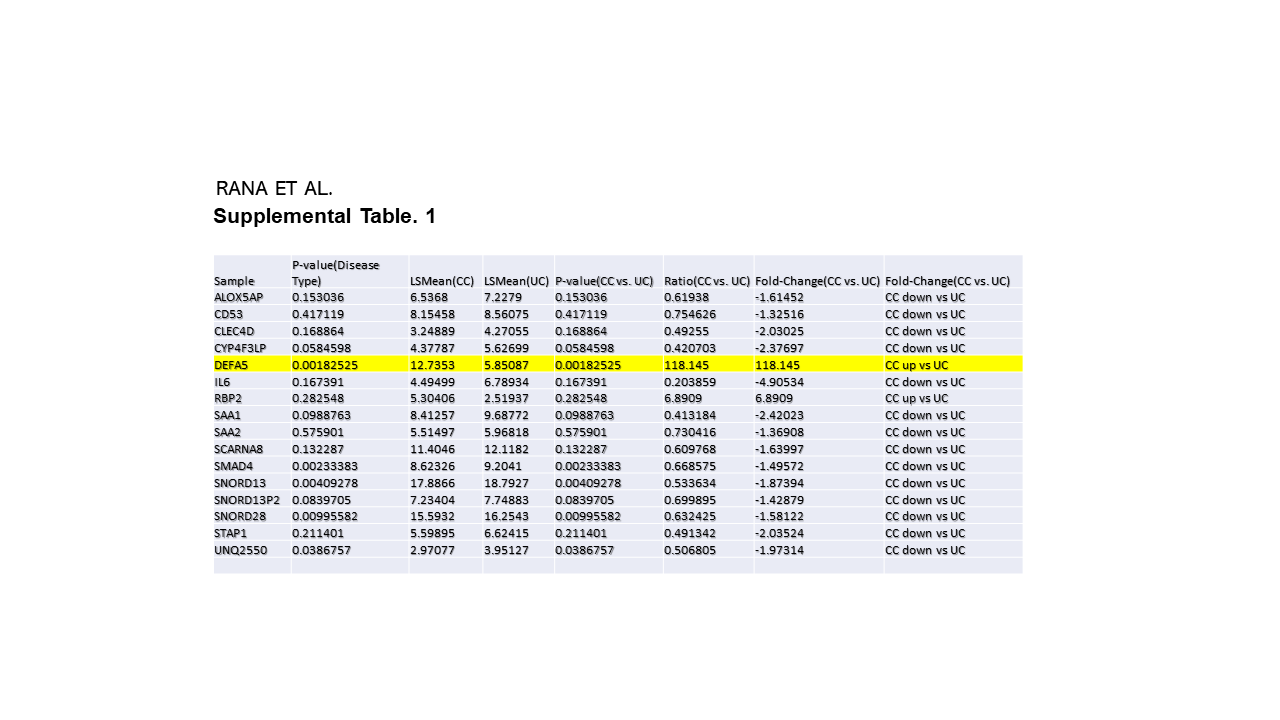

Supplement: S1 Table — Sixteen (#16) inflammatory genes were charged in this subset of samples. (TIF) [file pone.0246393.s002.tif]
